# Mineral Elements in the Raw Milk of Several Dairy Farms in the Province of Alberta

**DOI:** 10.3390/foods8080345

**Published:** 2019-08-14

**Authors:** Grzegorz Zwierzchowski, Burim N. Ametaj

**Affiliations:** 1 Department of Agricultural, Food and Nutritional Science, University of Alberta, Edmonton, AB T6G 2P5, Canada; 2 Faculty of Biology and Biotechnology, University of Warmia and Mazury, ul. Oczapowskiego 5, 10-719 Olsztyn, Poland

**Keywords:** whole raw milk, milk minerals, production system, heavy metals, food safety

## Abstract

The objective of this study was to determine the concentrations of 20 minerals in the whole raw milk from Holstein dairy cows in the province of Alberta, Canada. A total of 156 milk samples were collected from 26 dairy farms (*n* = 6 per farm) and analyzed with inductively coupled plasma mass spectrometry (ICP-MS) for five macrominerals (Ca, Mg, P, K, and Na), ten microminerals (Bo, Co, Cu, Fe, Mn, Mo, Ru, Se, St, and Zn), and five heavy metals (Al, As, Cd, Cr, and Pb). Calculated means were compared with their recommended daily intakes (RDIs) or minimal risk levels (MRLs) obtained from several food safety agencies and with data obtained from a world meta-analytical study we conducted previously. Results of the present study showed differences in the concentrations of multiple minerals between the Alberta farms involved and world averages (WA) and within Alberta farms. Concentrations of macrominerals, including Ca, Mg, P, K, and Na, in the raw milk were greater in Alberta dairy farms than the WA (*p* < 00.5; except Ca). Of note, concentrations of Ca showed the highest variability among Alberta farms, with 11 farms having lower milk Ca than WA. The other macrominerals were higher than WA in more than 88% of Alberta farms. Data demonstrated that concentrations of microminerals, including Co, Cu, Fe, Mn, and Mo, in Alberta raw milk were lower compared with WA (*p* < 0.05). Selenium was the only element in raw milk that was found to have higher concentrations in all farms in Alberta vs. WA. High variability was observed for B, Sr, and Zn, which were lower in multiple locations around the province. Concentrations of heavy metals in the Alberta raw milk, including Al, As, Cd, and Pb, were lower than WA, whereas concentrations of Cr were higher. Most importantly, all heavy metals were below their respective MRLs in all analyzed samples. Overall, data from this study showed that raw milk from Holstein dairy cows in Alberta has concentrations of most mineral elements below their MRLs and some of them different from WA. Of note, although concentrations of Se and Zn in the raw milk were higher in Alberta compared with WA, their concentrations were still below their respective MRLs.

## 1. Introduction

Milk contains essential components with different biological functions, including nutrients such as amino acids, fatty acids, proteins, vitamins, and minerals as well as immunoglobulins and antimicrobials, but at the same time, it might contain potentially harmful amounts of metals. The composition of dairy products reflects neonatal requirements for growth and development and immunological protection. For this reason, milk consists of significant amounts of proteins, fat, fat- and water-soluble vitamins, and minerals [1,2,3]. 

Milk composition has been reported to be associated with the breed of cattle, diet provided to dairy cows, minerals and vitamins supplementation, production management system, soil, and the use of pesticides or insecticides to the feedstuff provided to dairy cows [4]. Those factors, in combination with the growing environment pollution, agricultural, and urban emissions, are some of the reasons that milk and dairy products may contain toxic chemical pollutants [5]. Heavy metals such as cadmium (Cd), chromium (Cr), lead (Pb), or nickel (Ni) accumulate in plant materials and consequently pass into the milk and milk products. Recently, we reported that concentrations of two heavy metals including Cd in the raw milk from several areas of India, Nigeria, Palestine, and Slovakia, and Pb from several areas of Brazil, Croatia, Egypt, India, Mexico, Nigeria, Palestine, Serbia, and Turkey were above their MRLs (minimum risk levels) [6]. However, to the best of our knowledge, there is no data published with regards to content of milk metals in the raw milk of dairy cows in Canada. Consumption of milk surpassing the MRLs for heavy metals may lead to replacement of essential metal ions in the body and trigger harmful health effects [7]. Therefore, measurement of metals in the milk is of utmost importance to safeguard this nutritious product from potential contamination with heavy metals [8]. 

Improvements in the identification and quantification of metals in liquids by mass spectrometry (MS) has made it possible to accurately measure concentrations of a multitude of metals in milk. To date, the most commonly used analytical procedure for quantification of minerals involves inductively coupled plasma mass spectroscopy (ICP-MS) or inductively coupled plasma atomic-emission spectroscopy (ICP-AES) [1,9]. It is obvious that more research needs to be conducted in this field to address consumers’ concerns with regards to mineral content of milk, especially the concentrations of heavy metals. Indeed, in a recent meta-analytical study, we investigated mineral status of dairy cow raw milk from 37 countries and three types of production systems, including conventional (CONV), organic, and commercial (COMM) or retail store. Data showed that the organic milk has the lowest concentrations of the reported heavy metals versus the conventional and retail store milk [6]. Moreover, several heavy metals were found to be present in concentrations higher than the MRLs for the identified metals (Pb, As, and Hg) in several countries and areas of the world. To the best of our knowledge, there are no published data with regards to concentration of metals in the raw milk of dairy cows in Alberta or Canada. Therefore, the objective of this study was to quantify major macro- and microminerals as well as several heavy metals in the raw milk of Holstein dairy cows from 26 dairy farms in the province of Alberta. The aim is to compare results of this study with data collected and reported in our recently published meta-analytical study related to raw milk metals reported from different countries and with regards to the management system (organic, conventional, or retail store milk world-wide) [6]. The second aim is to provide important information to dairy producers in Alberta, Canada and beyond, as well as to nutritionists, nutrition advisors or consultants, food safety specialists, and different consumer groups with regards to milk metal content. 

## 2. Materials and Methods 

### 2.1. Milk Sample Collection

A total of 156 samples of raw milk were collected from 26 different farms in Alberta, Canada, from the northern, central, and southern regions of the province, in regions surrounding the cities of Edmonton, Red Deer, and Lethbridge (i.e., Northern, Central, and Southern Alberta areas, respectively). These three regions have different feeding regimens, with the northern region feeding mostly barley grain and forage, central feeding both corn and barley grain and forage, and the southern one feeding mostly corn grain and silage. Of note, we have not conducted soil or feedstuff mineral analyses in this study. Each of the 26 dairy farms had six samples analyzed. The study was conducted with the consent and agreement between Alberta Milk and CanWest DHI Lab and it was funded by Alberta Milk (dairy industry in Alberta) and the University of Alberta. All milk samples analyzed were provided during 2015 by CanWest DHI Laboratory in Edmonton, Alberta, Canada. 

### 2.2. Mineral Identification and Quantification

Raw milk samples were analyzed for 20 minerals by ICP-MS. Mineral elements and quantified included macrominerals calcium (Ca), magnesium (Mg), phosphorus (P), potassium (K), and sodium (Na); microminerals boron (B), iron (Fe), manganese (Mn), cobalt (Co), copper (Cu), zinc (Zn), selenium (Se), rubidium (Rb), strontium (Sr), and molybdenum (Mo); and heavy metals aluminum (Al), arsenic (As), cadmium (Cd), chromium (Cr), and lead (Pb). 

One mL of raw milk was mixed with 0.1 mL of HNO_3_, 0.1 mL of internal standards, and 8.8 mL of distilled water. Samples were then homogenized and analyzed with flow rate of 1 mL/min. Thirty-five sweeps/readings per sample were conducted with one reading per replicate and three replicates. Dwell times were 10 ms for Na, Al, K, Cu, Zn, and Sr, and 20 ms for the other metals, except for Se (150 ms). The integration times were the dwell time multiplied by the number of sweeps (in this case, it was 35). Therefore, integration times used were 350 ms for Al, K, Cu, Zn, and Sr; 5250 ms for Se, and 700 ms for the other elements. The final results are the average of three replicates. The instrument used to conduct the measurements was Perkin Elmer’s Elan 6000 with four points of calibration curve (0, 0.25, 0.50, 1.00 ppm for Na, Ca, Mg, Fe, K, and P as well as 0, 0.005, 0.010, 0.020 ppm for the rest of the elements).

### 2.3. Statistical Analysis

For each mineral, weighted mean and its associated variance, standard deviation, and standard error were calculated using the following models:

$$X_{w}=\frac{n_{1}}{N}x_{1}+\frac{n_{2}}{N}x_{2}+\frac{n_{r}}{N}x_{r}\;\sigma_{w}^{2}=\frac{\left(n_{1}-1\right)}{N-r}\sigma_{1}^{2}+\frac{\left(n_{2}-1\right)}{N-r}\sigma_{2}^{2}+\frac{\left(n_{r}-1\right)}{N-r}\sigma_{r}^{2}\;sd_{w}=\sqrt{\sigma_{w}^{2}}\;se_{w}=\frac{sd_{w}}{\sqrt{N}}$$

where:

*X_w_* is weighted mean
$\sigma_{w}^{2}$ is weighted variance
*sd_w_* is standard deviation of weighted mean
*se_w_* is standard error of weighted mean
*x*_1_, *x*_2_, *x_r_* are the individual samples
*n*_1_, *n*_2_, *n_r_* are individual sample sizes of *x*_1_, *x*_2_, *x_r_*, respectively
*N* is total sample size (*N = n*_1_ *+ n*_2_ *+ n_r_*)
$\sigma_{1}^{2},\;\sigma_{2}^{2},\;\sigma_{r}^{2}\;$ are individual sample variances
*r* is number of observations for each element

To quantify the differences in concentration of each analyzed variable in different types of milk, all data were subjected to modeling analysis using *t*-test for means with unequal variances (Alberta vs. COMM (Commercial), Alberta vs. CONV (Conventional)):

$$t=\frac{\left(x_{1}-x_{2}\right)-0}{\sqrt{\frac{\sigma_{1}^{2}}{n_{1}}+\frac{\sigma_{2}^{2}}{n_{2}}}}$$

where:

*x*_1_, *x*_2_ are weighted means
$\sigma_{1}^{2},\;\sigma_{2}^{2}\;\mathrm{are}\;\mathrm{weighted}\;\mathrm{variances}$
*n*_1_, *n*_2_ are numbers of observations

Subsequently degrees of freedom (*df*) were calculated using following equation:

$$df=\frac{{\left(\frac{\sigma_{1}^{2}}{n_{1}}+\frac{\sigma_{2}^{2}}{n_{2}}\right)}^{2}}{\frac{{\left(\frac{\sigma_{1}^{2}}{n_{1}}\right)}^{2}}{n_{1}-1}+\frac{{\left(\frac{\sigma_{2}^{2}}{n_{2}}\right)}^{2}}{n_{2}-1}}$$

where:

$\sigma_{1}^{2},\;\sigma_{2}^{2}\;$ are weighted variances
*n*_1_, *n*_2_, are numbers of observations

Results of each *t*-test were compared with critical values of student t distributions table at *p* ≤ 0.05. Calculated means for each mineral were compared with their respective recommended daily intake values (RDIs for macrominerals) or minimum risk levels (for heavy metals) obtained from the World Health Organization (WHO), the USA Agency for Toxic Substances and Disease Registry (ATSDR), Food and Drug Administration (FDA) daily values, European Food Safety Authority (EFSA), and Australian National Health and Medical Research Council [10,11,12,13]. 

## 3. Results

It should be noted that all the data presented in this study with regards to milk minerals in the 26 dairy farms in Alberta are compared with the data we generated and published in a previous meta-analytical study [6]. The data from this study will also be compared with mineral content of the world raw milk originating from world CONV dairy farms and from world COMM or retail stores. Of note, the present data were not available by the time the meta-analytical study was conducted and published. In fact, there was a scarcity of data for mineral content of the raw milk in Alberta and Canada. To the best of our knowledge, this is the first complete report of mineral elements in the raw milk of Holstein dairy cows in the province of Alberta, Canada. 

**Figure 1:**
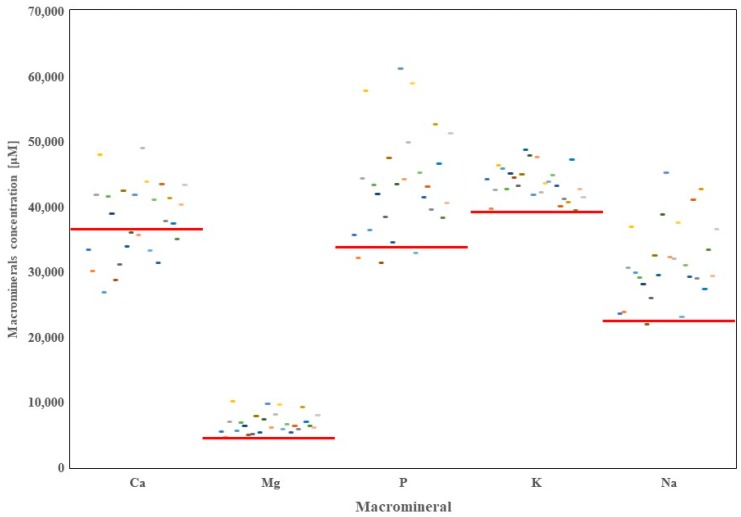
Distribution of macrominerals in raw milk samples from 26 dairy farms in Alberta with regards to world averages (red line).

**Figure 2:**
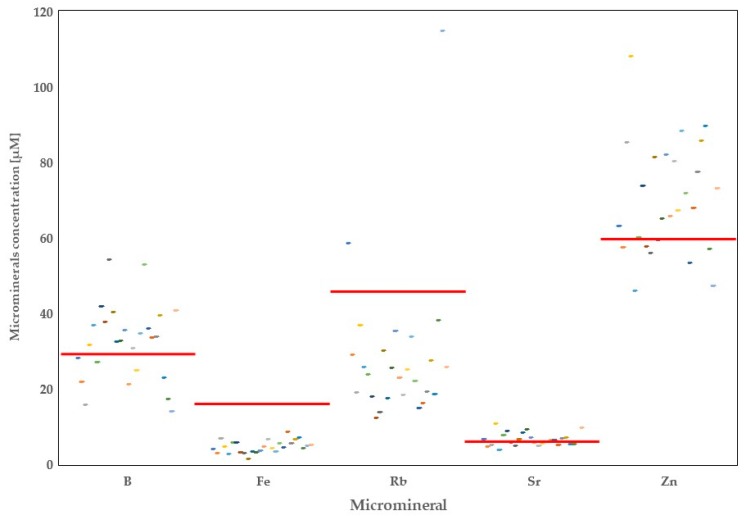
Distribution of microminerals (boron, iron, rubidium, strontium, and zinc) in Alberta milk samples with regards to world averages (red line).

**Figure 3:**
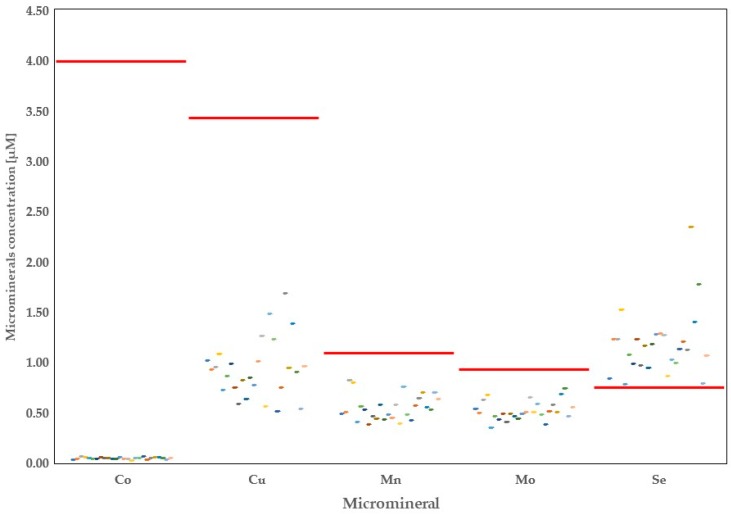
Distribution of microminerals (cobalt, copper, manganese, molybdenum, and selenium) in Alberta milk samples with regards to world averages (red line).

**Figure 4:**
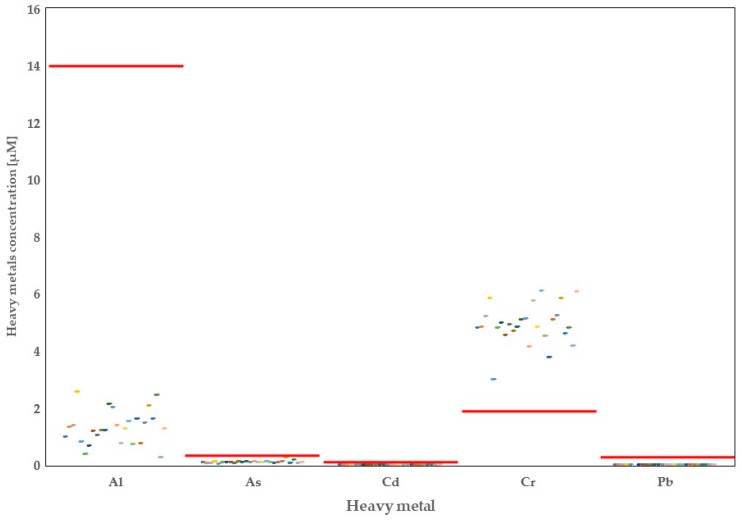
Distribution of heavy metals (aluminium, arsenic, cadmium, chromium, and lead) in Alberta milk samples with regards to world averages (red line).

A total of 20 minerals were quantified in the raw milk originating from 26 dairy farms in the province of Alberta. Thorough analysis of the data showed high variability in the concentrations of mineral elements depending on the region of origin (Figure 1, Figure 2, Figure 3 and Figure 4). 

Detailed analysis of minerals distribution in the raw milk showed high homogeneity of mineral content in different regions of Alberta. However, milk samples collected in the Edmonton area (Northern region) had lower B but higher As and Rb compared with the Lethbridge area (Southern region) (*p* < 0.05). Additionally, milk from the Red Deer area (Central region) contained lower Rb than milk from the Northern part (Edmonton area) of the province (*p* < 0.05) (Table 1).

Overall, concentrations of Ca in the raw milk of Alberta dairy farms was 37,980 μM, which was greater than the average concentration of Ca reported for the raw milk worldwide (36,632 μM) (Table 2); however, the difference reached no significance. 

On the contrary, the raw milk in Alberta dairy farms contained significantly higher amounts of Ca compared with COMM milk WA (Table 3). Forty-six percent of the dairy farms included in this study showed concentrations of Ca above the WA, with two farms exceeding the world concentration of 45,000 μM. Milk samples collected in the region of Coaldale contained the lowermost amounts of Ca (26,904 μM), whereas in Lomond, the raw milk contained the highest Ca concentration at 48,993 μM. 

The average concentration of Mg in the Alberta raw milk reached 6803 μM, which is 142% of the average concentration of Mg in the raw milk found in our previous worldwide report (4792 μM) (*p* < 0.05). Additionally, Alberta raw milk was significantly richer in Mg than the COMM or CONV milk available worldwide (Table 3; *p* < 0.05). The lowest concentration of Mg in the raw milk in Alberta was found in the milk deriving from Athabasca area (4625 μM), whereas the greatest amounts of Mg were found in the raw milk deriving from dairy farms in the Calmar area (10,166 μM). Of note, only the raw milk samples from Athabasca had lower concentrations of Mg compared to WA of 4855 μM.

In the present study, concentrations of inorganic P in the raw milk ranged from 31,393 μM (Duchess area) to 61,142 μM (Innisfail area). The calculated mean for Alberta raw milk samples for P reached 43,545 μM, and it was 28% higher than the WA concentration of 34,093 μM (*p* < 0.05). Additionally, it was found that the average concentrations of P in the raw milk from Alberta dairy farms were greater compared with concentrations of P in the COMM or CONV raw milk samples reported worldwide (Table 3; *p* < 0.05). Of note, only dairy farms located in the regions of Athabasca, Duchess, and Leduc had P concentrations below the WA. 

Potassium in the raw milk of Alberta dairy farms was at an average level of 43,672 μM, which is 11% greater than WA levels of K reported (39,332 μM; *p* < 0.05). Milk samples from Alberta contained higher concentrations of K compared with COMM or CONV milk samples worldwide (Table 3; *p* < 0.05). The lowest K level reported in our study was in milk originating from Rocky Mountain House (39,396 μM), whereas the highest concentrations of K were found in raw milk collected near Gem (48,730 μM). Of note, concentrations of K in all farms in Alberta were above the WA. 

The mean concentration of Na in the Alberta raw milk was 31,565 μM and it was greater than the means calculated for COMM and CONV milk samples worldwide (Table 3; *p* < 0.05). The lowest Na concentration was found in the milk collected from Duchess dairy farms (21,053 μM). Additionally, this region was the only one with concentrations of Na in the raw milk below the WA (22,516 μM). In the rest of the Alberta dairy farms, concentrations of Na were higher, reaching 201% of the WA as was the case with Innisfail region with concentrations of Na reaching 45,160 μM. 

Besides the macrominerals, 11 microminerals and trace elements were quantified in this study. The minimum level of B found in the raw milk samples of Alberta dairy farms was 14.01 μM (in the Tofield region), whereas the maximum concentrations were found to be 54.26 μM (in the Etzikom area). The average concentration of B in the 26 dairy farms in Alberta reached 32.25 μM, or 110% of the B concentration of the world raw milk; however, the difference reached no significance. Moreover, Alberta raw milk samples contained lower amounts of B compared with world’s CONV but higher than the COMM milk. In 17 farms, we found higher B concentrations when comparing to the reported WA levels of B (Figure 2). 

The mean concentration of Fe in the raw milk of Alberta dairy farms was 3.5-fold lower than the WA (4.67 μM vs. 16.63 μM). Comparison of the calculated mean concentration for Fe in the raw milk of Alberta with the concentrations of the COMM and CONV milk samples indicated that both types of milk contained more Fe than Alberta milk (Table 2). The greatest amount of Fe in Alberta dairy farms was 8.55 μM, found in the raw milk samples originating from the Magrath region. Additionally, milk samples originating from Fort MacLeod had very low Fe concentrations at 1.49 μM. 

Rubidium was significantly lower in Alberta milk samples compared with the average mean calculated for milk analyzed in other countries (*p* < 0.05). However, concentration of Rb calculated for Alberta was greater than the one calculated for COMM milk worldwide. On the other hand, milk Rb in Alberta was 3.5-fold less than the Rb concentration found in CONV milk worldwide (Table 3, *p* < 0.05). Of note, in 24 farms in Alberta, we found that concentrations of Rb were below the WA (28.56 μM compared with 47.05 μM). Only milk samples collected in the regions of Athabasca and Tofield exceeded that WA mean (58.51 μM, and 114.92 μM, respectively). The lowermost concentration of Rb was found in raw milk sample collected in the Etzikom region. 

Concentrations of St and Zn in the milk varied among samples collected in Alberta. Both minerals reached average means similar to those of WAs (Table 2). The lowest milk means for Sr and Zn were found in the Athabasca (Sr; 4.59 μM) and Coaldale (Zn; 45.89 μM) regions, whereas milk collected from Calmar area contained higher amounts of both minerals (10.77 μM, and 108.14 μM, respectively). Additionally, lower amounts of Sr were found in the COMM milk, but not in the CONV milk samples compared to Alberta (*p* < 0.05). On the contrary, Zn was lower in both production systems, including COMM and CONV milk, when compared with Alberta data. 

Average concentration of Co in the raw milk of Alberta dairy cows was around 1% of the WA (0.04 μM vs. 3.99 μM, respectively) (Figure 3). Moreover, its concentration was significantly lower compared with worldwide COMM and CONV (Table 2; *p* < 0.05) raw milk. The minimal level of Co found in the Alberta raw milk was 0.02 μM in the area of Leduc, whereas the highest concentration of this mineral were found in the milk samples collected in the area of Bowden (0.06 μM). 

Copper ranged from 0.51 μM (Linden region) to 1.68 μM (Millicent region), with the average of 0.93 μM, which represents 27% of Cu found in the milk samples worldwide. Importantly, all farms in Alberta had concentrations of Cu in the milk below the WA. Moreover, Cu concentration in the Alberta milk samples was nearly four-fold lower compared with CONV and COMM milk samples reported for other countries (Table 3; *p* < 0.05). 

The average means of Mn and Mo concentrations found in the Alberta cows’ milk were 0.55 and 0.52 μM, respectively. Milk in Alberta contained lower amounts of both minerals versus milk samples in other countries (Table 2; *p* < 0.05). Manganese ranged from 0.38 μM (Duchess area) to 0.82 μM in Bowden, whereas Mo ranged from 0.35 μM in Coaldale to 0.74 μM in Rocky Mountain House. Comparing means reported in the literature for both minerals, Mn and Mo were lower in Alberta milk samples compared with those of COMM and CONV milk worldwide (*p* < 0.05). 

Alberta milk contained greater amounts of Se compared with other dairy farms worldwide (1.18 μM vs. 0.77 μM). However, detailed analysis showed lower milk Se in the Alberta farms compared with the COMM milk but it was higher compared with CONV milk samples (Table 3). In all 26 farms analyzed, milk Se was found to be above the WA, with the lowest concentrations found in Coaldale milk (0.78 μM). Milk collected in Olds area contained more than three-fold (2.34 μM) the average amount of Se in the raw milk of dairy farms around the globe.

Overall, in the presented study, we examined concentrations of several important heavy metals in the raw milk samples collected from 26 dairy farms in the province of Alberta (Figure 4). It should be noted that all heavy metals determined in the Alberta raw milk were below their respective minimum risk levels (MRL’s). It should be noted that while concentrations of Al, As, Cd, and Pb were below their WAs, the concentration of Cr was above the world average. The lowest concentrations for heavy metals were observed in the areas of Tofield (Al), Coaldale (As and Cr), Leduc (Cd), and Drumheller (Pb). Most importantly, in all milk samples analyzed in the Drumheller area, Pb levels were below the limit of detection for this element. On the contrary, Al was highest in the Calmar area, As and Cd in the Olds area, Cr in the Leduc area, and Pb in Picture Butte area. 

## 4. Discussions

In a recent research article [6], we showed large variability with regards to concentrations of major milk minerals worldwide. Moreover, in some countries, our meta-analytical analysis showed that concentrations of selected heavy metals (i.e., Cd and Pb) were above their MRLs in the raw milk samples. Since the physiological and health significance of milk minerals was covered extensively in the aforementioned article, in the present study we will focus our discussion on the raw milk mineral differences between Canada vs. the world and on the mineral nutritional quality of Alberta raw milk. For this purpose, we compared the total amount of each milk mineral with the RDIs or MRLs for each analyzed mineral considering an intake of 300 mL of milk in a diet, based on 2000 calories per day (Table 4). 

In order to eliminate the influence of season, all samples were collected during a similar period of time (approximately 3 months). It has to be noted that since 95% of dairy cows in Alberta are represented by the Holstein breed [14], any fluctuations in the concentrations of milk minerals are unlikely to be related to genetic factors. 

With regards to the group of macrominerals, raw milk from the province of Alberta, Canada contained, as an average, higher concentrations of Ca, Mg, P, K, and Na compared with the respective average concentrations of those minerals in the world raw milk. Concentrations of macrominerals in the raw milk of Alberta should be linked with the dietary composition, and with the mineral content of soil, plants, and water in dairy farms. Several studies have shown that milk Ca is greater in the raw milk containing high concentration of proteins [15,16,17]. Several other labs have demonstrated that excessive mineral supplementation increases concentrations of Mg and P in the raw milk [18,19]. Moreover, K in the raw milk is high in several developed countries in relation with the excessive utilization of artificial fertilizers and slurry for crop development [20,21]. Furthermore, Na in the milk is believed to represent the osmoregulatory status of each individual cow [22,23]. It should be pointed out that a previous research article reported that water offered to cows influences the content of macrominerals in Alberta raw milk [24]. Results of our study show that Alberta raw milk should be considered as a good source of macrominerals. Based on our data, cow’s raw milk in Alberta covers approximately 40% of the RDIs for Ca and P, almost 15% of the RDIs for Mg and K, and less than 10% of RDI for Na. Since milk is not the only source of macrominerals in the diet, it might be considered as a complementary dietary supplement for minerals. 

Milk trace elements are important components of the organism’s metalloenzyme complexes [25]. In this study, we investigated ten different microminerals. Boron is known as a milk aqueous phase component, strongly related to its content in the environment [1]. Our research confirms these reports. In our study, samples collected in a radius of approximately 40 km contained significantly different amounts of this mineral (52.97 µM for raw milk originating from Lethbridge area vs. 31.13 µM for the raw milk coming from Magrath and Picture Coaldale area). Interestingly, according to previous research [26], it was expected that the greatest concentrations of B would be in the milk samples collected from the Northern area of the province. However, this was not the case in this study, as the milk samples collected from the Southern part of the province exhibited greater concentrations of B. Boron is considered an essential nutrient for plants’ growth. Importantly, greater amounts of B were found in forages and grains but lower in the barley grain [27]. This is in line with our findings, since there are differences in the feedstuff offered to the dairy cows in various regions of Alberta where the southern region feeds mostly corn grain and forage (or silage); the central part of the province feeds more of a mixture of corn and barley grain and forage (or silage) and the northern region feeds mostly barley grain and forage. It should be noted that presence of B in the environment is anthropogenic (influenced by humans); therefore, increased B in the raw milk samples collected from the Southern part of Alberta might be related to application of fertilizers or herbicides containing boron, or use of waste water for irrigation [28]. It should be noted that Alberta raw milk delivers only 0.52% of the RDI for B. 

According to Patra et al. [29], Co in the raw milk increases with increasing concentrations of Pb in the milk. Our findings do not confirm these observations since Co and Pb showed independent distribution. For example, in the milk coming from Picture Butte area, we found higher Pb than in any other area under study, whereas concentrations of Co showed similarity among various areas including Picture Butte. Therefore, any conclusions regarding milk Co in the Alberta raw milk should be treated with caution and requires further investigation. In terms of nutrition, milk from Alberta dairy farms provides less than 4% of the RDI for this mineral. 

It has been known for a long time that intakes of Cu and Zn are strongly antagonistic. Zinc and Cu compete and/or inhibit each other at the level of intestinal epithelial cells in relation with concentration of each of the elements in the intestinal lumen [30]. Furthermore, it should be noted that although these elements are considered essential metals, excessive intake may cause adverse effects [31]. Enhanced concentration of one of the elements in the milk consequently limits concentration of the other element [32]. Data from our meta-analytical study [6] and from the present study support this antagonism. In milk from Alberta we found greater concentrations of Zn in the raw milk versus WA, which probably resulted in significantly lower concentrations of Cu in the same milk. Overall, Alberta milk covers less than 1% of the RDI for Cu and 9.16% of the RDI for Zn. 

Milk Fe was previously shown to be positively correlated with its amounts in the water offered to livestock animals [33]. Linn [24] showed high amounts of Fe in Alberta water; however, we found lower Fe in the raw milk samples in Alberta compared with the WA. These findings indicate that there might be some other unknown factor(s) influencing concentrations of Fe in Alberta raw milk [23,34]. Regarding Fe RDI, an adult needs to consume around 15 mg of Fe per day. According to our calculations, raw milk Fe in Alberta can cover less than 1% of this requirement. Therefore, raw Alberta milk cannot be considered as a good source of Fe in the diet. 

The results of our study also showed lower Mn in Alberta raw milk compared with other studies. Manganese has been reported to be increased in the milk of dairy cows in areas surrounding heavy industry facilities or in polluted areas [32]. In Alberta, however, the main factor possibly affecting concentration of Mn in the raw milk is genetics. As previously demonstrated, Holstein breed of cows has lower Mn in the milk versus the other breeds of cattle [35]. Our study is in agreement with this previously reported finding. The very low concentrations of Mn in Alberta raw milk can provide less than 0.2% of the RDI for Mn. Therefore, it can be concluded that milk in itself is an insufficient source for this mineral. 

Milk Mo is associated mostly with milk-fat-globule membrane. Therefore, the dietary bioavailability of this mineral is believed to be a limiting factor for Mo concentration in the milk [21]. In the raw milk of Alberta, Mo varied from 0.35 to 0.74 µM, which represents 36% to 77% of the WA. Lower concentrations of this mineral in the milk can be considered an advantage of the Alberta milk, since a glass of milk provides less than 20% of its RDI. Due to negative implication of excessive intakes of Mo on reproductive functions of males [36,37], lower amounts of Mo in the Alberta raw milk make it a safe product to consume [38].

Rubidium is not considered an essential mineral, although it is present in almost all animal tissues. This is related to its good solubility in the water, which results in its accumulation in plants and animal tissues. However, it should be pointed out that the precise role of Rb in the metabolism of animals and cows remains unclear. Nevertheless, according to several reports, no harmful effects of excessive Rb intake have been reported [39,40,41,42,43]. In this study, we found lower concentrations of Rb in Alberta raw milk compared with the WA. However, in milk samples collected from Tofield and Athabasca areas located in the Northern part of the province, milk Rb was significantly higher than in other milk samples from Alberta. On the other hand, out of two samples collected from the Tofield and Athabasca areas, one contained half as much Rb as the other one. It is known that Rb has good bioaccumulation properties, and it is abundant in the areas rich in granite or illite clays, which have been reported as the main clay types in Alberta [44]. Due to there being only a few studies conducted on the Rb impact on human health, there is currently no RDI established for this element [45].

According to our data, Alberta’s raw milk contains 54% more Se than the calculated WA. Thus, a glass of Alberta raw milk covers nearly 80% of the RDI for this mineral. As discussed in our previous meta-analytical study [6], concentrations of Se in the raw milk are dependent on the intake from the diet. In rural areas, where soils are poor in Se, this mineral is lower in the milk. 

This tendency is also true for Alberta, where soils rich in selenium, otherwise known as seleniferous soils, are very common [46]. Of note, excessive amounts of Se intake might cause various adverse effects [47,48]. Given that the raw milk in Alberta has lower concentrations of Se than its RDI (80%), it should be concluded that Alberta milk is safe for consumption with regards to Se content.

Due to its chemical properties and similarity to Ca, Sr is easily absorbed by human body; however, this is true for the vaporized form of Sr. When ingested, only a small portion of Sr is absorbed into the blood circulation [49,50]. Animals secrete Sr via body fluids such as urine, sweat, or milk. Most Sr is absorbed by plants from soil; however, it has been shown that Sr content in Alberta soils may vary significantly [51]. This mineral is not of concern for Alberta consumers, because Alberta milk covers only 4.27% of the RDI for this metal, and does not differ from the World mean concentration of Sr in the raw milk. 

The group of minerals known as heavy metals are naturally occurring minerals with possible harmful effects in case of excessive ingestion [52]. In our study, we analyzed presence of five heavy metals, including Al, As, Cd, Cr, and Pb. Heavy metals are present in the raw milk samples analyzed in Alberta dairy farms; however, concentrations of those metals are very low and below the MRLs for human intake. The excessive intake of heavy metals above the recommended levels is harmful for human health. Lead (Pb) and cadmium (Cd) are the most toxic elements for humans and cattle health because of their cumulative effect [5,29]. The intake of 300 mL/d of Alberta raw milk represent only 8.85 and 5.62% of the safety recommendation for these heavy metals, respectively. 

An interesting finding of our study is that milk in the Northern part of Alberta contains greater As than raw milk collected at southern part of the province. Concentration of As is related to concentration of P due to the competition for adsorption sites on oxyhydroxide surfaces [53]. This observation is true in our study, where we found higher As but lower P in Northern part of Alberta, while the lowest As was accompanied with the highest P among province subregions. According to Lemay [54], higher As concentrations in Northern and Central parts of Alberta are related to the geology of the province. Additionally, some as might have anthropological origin, related to crop fertilizers or wood preservatives. The raw milk daily intake for as in raw milk in Alberta is 23.94%. It should be noted that concentrations of As were below its MRL; therefore, Alberta milk can be considered a safe product for human consumption. 

The other heavy metals analyzed show a raw milk daily intake around 0.51% for Al and 63.93% for Cr. Of note, only Cr^6+^ is considered a potential harmful metal. In our study, we analyzed the total content of Cr and not the valency differentiations. Therefore, any conclusions regarding this element or its high concentration in the Alberta raw milk warrants cautious interpretation and further investigation. Moreover, it must be pointed out that consumption of Cr is beneficial to a certain point, since it has been shown to play a role in improving glucose tolerance [55]. 

## 5. Conclusions

Overall, to the best of our knowledge, this is the first study analyzing raw milk minerals in dairy farms in the province of Alberta, Canada. Data from this study indicate that Alberta milk has a safe mineral content for human consumption. Alberta milk is a good source of Ca and P, covering more than 40% of their RDIs for both minerals. On the other hand, Alberta raw milk offers lower than WA amounts but normal amounts of Mg, P, and Na. Considering consumption of other food sources in the diet, this suggests that Alberta raw milk provides substantial amounts for these minerals. Regarding average concentrations of microminerals and trace elements in the raw milk of Alberta dairy cows, greater concentrations of Se and Zn were found in the raw milk compared with the world CONV milk. Nevertheless, the calculated means for those two metals still were below their respective MRLs. The most important conclusion drawn from this study is that concentrations of heavy metals in the Alberta raw milk are significantly lower than the calculated means of all those elements compared with the WA. Alberta raw milk had high Cr content, albeit within its RDI, but it must be noted that only Cr^6+^ is considered harmful to humans and this study cannot conclude whether Cr^6+^ was high. Further investigation needs to determine the type of Cr valency contained in Alberta milk.

There were very few differences among the three regions of Alberta (Northern, Central, and Southern) with regards to mineral content of raw milk, including greater concentrations As and Rb and lower concentrations of B in the Northern region vs. the Central and Southern which might be related to the soil composition, origin of grain and forages, as well as water sources and mineral supplements. 

## Figures and Tables

**Table 1:** Concentration of macro- and microminerals as well as heavy metals in the raw milk of dairy cows in subregions of Alberta, Canada.

**Macromineral**	**Northern**	**SD**	***n***	**Central**	**SD**	***n***	**Southern**	**SD**	***n***	***p*-value**
Calcium	38,519	5941	48	37,078	7760	66	38,784	10,118	42	NS
Magnesium	7148	1353	48	6644	1448	66	6659	1348	42	NS
Phosphorus	44,072	8960	48	42,965	8134	66	43,852	9057	42	NS
Potassium	43,701	4942	48	43,417	6437	66	44,040	4835	42	NS
Sodium	31,200	6711	48	31,923	8169	66	31,420	6459	42	NS
**Micromineral**	**Northern**	**SD**	***n***	**Central**	**SD**	***n***	**Southern**	**SD**	***n***	***p*-value**
Boron	28.58 ^a^	5.13	48	30.74	7.91	65	38.80 ^b^	8.58	42	*p* < 0.05
Cobalt	0.04	0.01	48	0.05	0.02	66	0.04	0.01	42	NS
Copper	0.92	0.38	48	0.91	0.74	66	0.96	0.46	42	NS
Iron	4.05	1.59	48	4.90	3.72	65	5.06	1.93	42	NS
Manganese	0.58	0.18	48	0.55	0.20	66	0.50	0.18	42	NS
Molybdenum	0.53	0.09	48	0.51	0.18	66	0.51	0.12	42	NS
Rubidium	43.65 ^a^	12.12	48	22.60 ^b^	5.59	66	20.69 ^b^	3.10	42	*p* < 0.05
Selenium	1.06	0.16	48	1.31	0.83	66	1.11	0.26	42	NS
Strontium	7.31	1.79	48	6.76	2.31	66	5.31	1.99	42	NS
Zinc	71.25	16.25	48	68.88	20.24	66	73.90	25.23	40	NS
**Heavy metal**	**Northern**	**SD**	***n***	**Central**	**SD**	***n***	**Southern**	**SD**	***n***	***p*-value**
Aluminium	1.301	0.407	47	1.449	1.018	66	0.998	0.310	42	NS
Arsenic	0.106 ^a^	0.014	48	0.123	0.172	66	0.088 ^b^	0.017	42	*p* < 0.05
Cadmium	0.001	0.000	48	0.002	0.003	66	0.002	0.000	42	NS
Chromium	5.227	0.558	48	4.949	1.953	66	4.664	1.880	42	NS
Lead	0.003	0.002	48	0.004	0.003	66	0.004	0.001	42	NS

All values are expressed as µM concentration. ^a,b^ Least square means within a row lacking common superscripts differ at *p* < 0.05. NS, not significant.

**Table 2:** Concentration of macro- and microminerals as well as heavy metals in the raw milk of dairy cows in Alberta, Canada compared with the respective World averages.

**Macromineral**	**Canada**	**SD**	***n***	**Min**	**Max**	World *	**SD**	***n***	**Min**	**Country**	**Max**	**Country**	***p*-value**
Calcium	37,980	7327	156	26,904	47,955	36,632	5476	3345	2096	Czech Republic	242,016	Austria	NS
Magnesium	6803 ^a^	1248	156	4625	10,166	4792 ^b^	911	3,274	2350	Poland	38,272	Germany	*p* < 0.05
Phosphorus	43,545 ^a^	7873	156	31,394	61,148	34,093 ^b^	6544	818	13,691	Poland	271,230	Germany	*p* < 0.05
Potassium	43,672 ^a^	4920	156	39,396	48,730	39,332 ^b^	5939	3001	21,893	Czech Republic	371,355	Austria	*p* < 0.05
Sodium	31,565 ^a^	6453	156	21,954	45,160	22,516 ^b^	4900	2986	12,049	Czech Republic	130,492	Germany	*p* < 0.05
**Micromineral**	**Canada**	**SD**	***n***	**Min**	**Max**	World *	**SD**	***n***	**Min**	**Country**	**Max**	**Country**	***p*-value**
Boron	32.25	6.64	155	14.01	54.26	29.33	62.58	173	7.86	Poland	277.52	Germany	NS
Cobalt	0.04 ^a^	0.01	156	0.02	0.06	3.99 ^b^	1.95	511	<0.01	Spain	83.98	Germany	*p* < 0.05
Copper	0.93 ^a^	0.49	156	0.51	1.68	3.43 ^b^	9.08	3668	0.50	Poland	61.37	Slovakia	*p* < 0.05
Iron	4.67 ^a^	2.33	156	1.49	8.55	16.63 ^b^	24.93	2990	2.69	Czech Republic	155.42	India	*p* < 0.05
Manganese	0.55 ^a^	0.17	156	0.38	0.82	1.11 ^b^	0.42	1590	0.24	Czech Republic	6.92	China	*p* < 0.05
Molybdenum	0.52 ^a^	0.12	156	0.35	0.74	0.95 ^b^	0.28	274	0.11	Poland	3.86	Germany	*p* < 0.05
Rubidium	28.56 ^a^	7.14	156	13.84	58.51	47.05 ^b^	10.84	152	0.02	Japan	427.87	Austria	*p* < 0.05
Selenium	1.18	0.46	156	0.78	2.34	0.77	13.33	2240	0.07	Brazil	18.05	South Korea	NS
Strontium	6.54	1.84	156	4.59	10.77	6.65	1.09	283	0.05	Japan	35.61	Germany	NS
Zinc	70.02	18.85	156	45.89	108.14	64.04	29.13	3358	14.45	Saudi Arabia	688.18	Germany	NS
**Heavy metal**	**Canada**	**SD**	***n***	**Min**	**Max**	World *	***SD***	***n***	**Min**	**Country**	**Max**	**Country**	***p*-value**
Aluminum	1.28 ^a^	0.59	156	0.267	2.573	13.92 ^b^	9.17	564	1.89	Poland	275.28	Turkey	*p* < 0.05
Arsenic	0.11	0.09	156	0.056	0.281	0.39	1.09	648	0.01	South Korea	1.78	Italy	NS
Cadmium	<0.01	<0.01	156	<0.01	<0.01	0.17	0.76	2007	<0.01	Turkey	2.40	Slovakia	NS
Chromium	4.92 ^a^	1.44	156	3.017	6.096	1.91 ^b^	7.09	1032	0.07	Germany	33.78	Nigeria	*p* < 0.05
Lead	<0.01 ^a^	<0.01	156	<0.01	<0.01	0.34 ^b^	0.43	2218	<0.01	RSA	4.10	India	*p* < 0.05

All values are expressed as µM concentration; * World data adapted from Zwierzchowski & Ametaj, 2018 [6]; ^a–c^ Least square means within a row lacking common superscripts differ at *p* < 0.05.

**Table 3:** Concentration of macro- and microminerals as well as heavy metals in the raw milk of dairy cows in Alberta, Canada compared with the retail store milk (commercial) and farm milk (conventional) around the world.

**Macromineral**	**Canada**	**SD**	***n***	**Min**	**Max**	COM *	**SD**	***n***	**Min**	**Max**	CONV *	**SD**	***n***	**Min**	**Max**	***p*-value**
Calcium	37,980 ^a^	7327	156	26,904	47,955	31,819 ^b^	2054	461	2096	38,423	37,744 ^a^	5,885	2791	14,147	242,016	*p* < 0.05
Magnesium	6803 ^a^	1248	156	4625	10,166	4649 ^b^	467	479	4239	5144	4855 ^b^	977	2702	2350	38,272	*p* < 0.05
Phosphorus	43,545 ^a^	7873	156	31,394	61,148	28,285 ^b^	1726	32	27,058	29,383	35,954^c^	6,986	689	13,691	271,230	*p* < 0.05
Potassium	43,672 ^a^	4920	156	39,396	48,730	38,781 ^b^	2487	407	23,223	43,095	39,756 ^b^	5,817	2537	21,893	371,355	*p* < 0.05
Sodium	31,565 ^a^	6453	156	21,954	45,160	23,520 ^b^	3408	425	15,702	26,490	22,417 ^b^	5,109	2508	12,049	130,492	*p* < 0.05
**Micromineral**	**Canada**	**SD**	***n***	**Min**	**Max**	COM *	**SD**	**n**	**Min**	**Max**	CONV *	**SD**	***n***	**Min**	**Max**	***p*-value**
Boron	32.25 ^a^	6.64	155	14.01	54.26	10.58 ^b^	0.77	18	9.71	11.29	43.52	80.43	102	8.39	277.52	*p* < 0.05
Cobalt	0.04 ^a^	0.01	156	0.02	0.06	0.07 ^b^	0.01	87	<0.01	0.10	5.82^c^	2.34	349	<0.01	83.98	*p* < 0.05
Copper	0.93 ^a^	0.49	156	0.51	1.68	3.82 ^b^	3.10	573	1.08	27.22	3.43	9.95	2980	0.50	61.37	*p* < 0.05
Iron	4.67 ^a^	2.33	155	1.49	8.55	18.35 ^b^	9.60	522	3.04	147.31	16.62 ^b^	27.63	2353	2.69	155.42	*p* < 0.05
Manganese	0.55 ^a^	0.17	156	0.38	0.82	2.13 ^b^	0.72	167	0.24	6.92	1.04^c^	0.39	1308	0.31	4.79	*p* < 0.05
Molybdenum	0.52 ^a^	0.12	156	0.35	0.74	1.20 ^b^	0.44	55	0.26	1.98	0.96^c^	0.25	144	0.11	3.86	*p* < 0.05
Rubidium	28.56 ^a^	7.14	156	13.84	58.51	18.10 ^b^	0.02	96	0.02	27.27	96.69^c^	18.26	56	0.02	427.87	*p* < 0.05
Selenium	1.18 ^a^	0.46	156	0.78	2.34	2.99a	31.83	411	0.07	18.05	0.27 ^b^	0.12	1683	0.12	5.07	*p* < 0.05
Strontium	6.54 ^a^	1.84	156	4.59	10.77	4.29 ^b^	0.01	96	0.05	6.95	10.32^c^	1.59	134	0.06	35.61	*p* < 0.05
Zinc	70.02 ^a^	18.85	156	45.89	108.14	53.45 ^b^	8.32	550	41.6	72.71	67.33 ^a^	32.25	2689	14.45	688.18	*p* < 0.05
**Heavy metal**	**Canada**	**SD**	***n***	**Min**	**Max**	COM *	**SD**	***n***	**Min**	**Max**	CONV *	**SD**	***n***	**Min**	**Max**	***p*-value**
Aluminum	1.28 ^a^	0.59	155	0.27	2.57	NO DATA					15.13 ^b^	9.59	511	2.06	275.28	*p* <0.05
Arsenic	0.11 ^a^	0.09	156	0.06	0.28	0.05 ^b^	<0.01	87	0.01	0.23	0.49a	1.25	486	0.01	1.78	*p* < 0.05
Cadmium	<0.01 ^a^	<0.01	156	<0.01	<0.01	0.07 ^b^	0.05	145	0.01	0.91	0.19a	0.81	1747	0.00	2.40	*p* < 0.05
Chromium	4.92 ^a^	1.44	156	3.02	6.10	2.95 ^b^	0.65	161	0.09	7.02	1.85a	8.02	796	0.07	33.78	*p* < 0.05
Lead	<0.01 ^a^	<0.01	156	<0.01	<0.01	0.45 ^b^	0.31	203	0.02	1.11	0.34 ^b^	0.45	1900	0.00	4.10	*p* < 0.05

All values are expressed as µM concentration; * world data adapted from Zwierzchowski & Ametaj, 2018 [6]. ^a–c^ Least square means within a row lacking common superscripts differ at *p* < 0.05; COM—commercial milk, CONV—conventional milk.

**Table 4:** Concentration and the percentage of daily intake of micro- and macrominerals as well as heavy metals in the raw milk of Alberta, Canada versus recommended daily intakes (RDI).

**Macromineral**	**Total Concentration (mg/300 mL)**	**RDI ^1^** **(mg/day)**	**Intake from Milk ^3^ (%)**
Calcium	456.7	1000	45.67
Magnesium	49.59	350	14.17
Phosphorus	404.6	1000	40.46
Potassium	512.3	3500	14.64
Sodium	217.7	2400	9.07
**Micromineral**	**Total Concentration (µg/300 mL)**	**RDI ^1^** **(µg/day)**	**Intake from Milk ^3^ (%)**
Boron	104.6	20,000	0.52
Cobalt	0.8	19.85	3.83
Copper	17.7	2000	0.88
Iron	78.2	15,000	0.52
Manganese	9.0	5000	0.18
Molybdenum	14.9	75.00	19.83
Rubidium	643.9	ND	ND
Selenium	27.9	35.00	79.73
Strontium	171.9	4030	4.27
Zinc	1,373.8	15,000	9.16
**Heavy Metal**	**Total Concentration (µg/300 mL)**	**Toxic Dose ^2^ (µg/day)**	**Intake from Milk ^3^ (%)**
Aluminum	10.38	2020.30	0.51
Arsenic	2.43	10.14	23.94
Cadmium	0.05	0.97	5.62
Chromium	76.71	120.00	63.93
Lead	0.21	2.39	8.85

^1^ RDI = Recommendation daily intake or maximum values are based on values from the Australian National Health and Medical Research Council, the World Health Organization (WHO), and European Food Safety Authority (EFSA). ^2^ Toxic dose according to toxicological profiles by the Agency for Toxic Substances and Disease Registry, Atlanta, Georgia, US. ^3^ Daily intake was calculated based on a diet of 2000 calories for person of 4 or more years of age and using a milk intake of 300 mL with 3.7% of fat representing 192 calories or 0.96% of total of 2000 calories per day. ND, not detect.

## References

[1] Herwig N., Stephan K., Panne U., Pritzkow W., Vogl J. (2011). Multi–element screening in milk and feed by SF–ICP–MS. Food Chem..

[2] Roncada P., Piras C., Soggiu A., Turk R., Urbani A., Bonizzi L. (2012). Farm animal milk proteomics. J. Proteom..

[3] Miciński J., Zwierzchowski G., Kowalski I.M., Szarek J. (2013). Health–promoting properties of selected milk components. J. Elem..

[4] Ross E.M., Rajan M.P., Wesley S.G. (2012). Milk minerals in cow milk with special reference to elevated calcium and its radiological implications. Radiat. Prot. Environ..

[5] Suturović Z., Kravić S., Milanović S., Ðurović A., Brezo T. (2014). Determination of heavy metals in milk and fermented milk products by potentiometric stripping analysis with constant inverse current in the analytical step. Food Chem..

[6] Zwierzchowski G., Ametaj B.N. (2018). Minerals and heavy metals in the whole raw milk of dairy cows from different management systems and countries of origin: A meta-analytical study. J. Agric. Food Chem..

[7] Jan A.T., Azam M., Siddiqui K., Ali A., Choi I., Haq Q.M.R. (2015). Heavy metals and human health: Mechanistic insight into toxicity and counter defense system of antioxidants. Int. J. Mol. Sci..

[8] Forman J., Silverstein J. (2012). Organic Foods: Health and Environmental Advantages and Disadvantages. Pediatrics.

[9] Husáková L., Urbanová I., Šrámková J., Konečná M., Bohuslavová J. (2013). Multi–element analysis of milk by ICP-oa-TOF-MS after precipitation of calcium and proteins by oxalic and nitric acid. Talanta.

[10] Australian National Health and Medical Research Council Nutrient Reference Values for Australia and New Zealand. https://www.nrv.gov.au/nutrients.

[11] Agency for Toxic Substances and Disease Registry. https://www.atsdr.cdc.gov/.

[12] World Health Organization, Food and Agricultural Organization of the United Nations Vitamin and Mineral Requirements in Human Nutrition.

[13] European Food Safety Authority (2017). Summary Report on Dietary Reference Values for Nutrients.

[14] Alberta Agriculture and Forestry. https://www1.agric.gov.ab.ca/$department/deptdocs.nsf/all/beef1710#alberta.

[15] Woo S.J., Maeng Y.S. (1998). Nutrition of Milk and Dairy Products.

[16] Battestin L., Tacla R.M.B., Tiboni E.B., Freitas R.J.S., Stertz S.C. (2002). Análise de cálcio em diferentes tipos de bebidas. Acad. Vis..

[17] Yoo S.H., Kang S.B., Park J.H., Lee K.S., Kim J.M., Yoon S.S. (2013). Effect of heat-treat methods on the soluble calcium levels in the commercial milk products. Korean J. Food Sci. Anim. Resour..

[18] Withers P.J.A., Peel S., Mansbridge R.M., Chalmers A.C., Lane S.J. (1999). Transfers of phosphorus within three dairy farming systems receiving varying inputs in feeds and fertilizers. Nutr. Cycl. Agroecosyst..

[19] Gustafson G.M., Salomon E., Jonsson S. (2007). Barn balance calculations of Ca, Cu, K, Mg, Mn, N, P, S and Zn in a conventional and organic dairy farm in Sweden. Agric. Ecosyst. Environ..

[20] Gustafson G.M., Salomon E., Jonsson S., Steineck S. (2003). Fluxes of K, P, and Zn in a conventional and an organic dairy farming system through feed, animals, manure, and urine—A case study at Öjebyn, Sweden. Eur. J. Agron..

[21] Gabryszuk M., Słoniewski K., Sakowski T. (2008). Macro-and micro-elements in milk and hair of cows from conventional vs. organic farms. Anim. Sci. Pap. Rep..

[22] Shalit U., Maltz E., Silanikove N., Berman A. (1991). Water, sodium, potassium, and chlorine metabolism of dairy cows at the onset of lactation in hot weather. J. Dairy Sci..

[23] Qin L.Q., Wang X.P., Li W., Tong X., Tong W.J. (2009). The minerals and heavy metals in cow’s milk from China and Japan. J. Health Sci..

[24] Linn J. (2006). Impact of minerals in water on dairy cows. WCDS Adv. Dairy Technol..

[25] Smart M.E., Gudmundson J., Christensen D.A. (1981). Trace Mineral Deficiencies in Cattle: A Review. Can. Vet. J..

[26] Pawluk S., Bayrock L.A. (1969). Some Characteristics and Physical Properties of Alberta Tills.

[27] Card S., Cathcart J., Huang J. (2005). The Micronutrient and Trace Element Status of Crops Grown on the Alberta Soil Quality Benchmark Sites; AESA Soil Quality Monitoring Program.

[28] Alberta Environment and Parks (2015). Soil Remediation Guidelines for Boron: Environmental and Human Health.

[29] Patra R.C., Swarup D., Kumar P., Nandi D., Naresh R., Ali S.L. (2008). Milk trace elements in lactating cows environmentally exposed to higher level of lead and cadmium around different industrial units. Sci. Total Environ..

[30] Oestreicher P., Cousins R.J. (1985). Copper and zinc absorption in the rat: Mechanism of mutual antagonism. J. Nutr..

[31] Khaniki G.R.J. (2007). Chemical contaminants in milk and public health concerns: A review. Int. J. Dairy Sci..

[32] Król J., Litwińczuk Z., Brodziak A., Kędzierska-Matysek M. (2012). Content of selected essential and potentially toxic trace elements in milk of cows maintained in Eastern Poland. J. Elem..

[33] Mann G.R., Duncan S.E., Knowlton K.F., Dietrich A.D., O’Keefe S.F. (2013). Effects of mineral content of bovine drinking water: Does iron content affect milk quality?. J. Dairy Sci..

[34] Rodríguez E.M.R., Sanz Alaejos M., Díaz Romero C. (2001). Mineral Concentrations in Cow’s Milk from the Canary Island. J. Food Comp. Anal..

[35] Hermansen J.E., Badsberg J.H., Kristensen T., Gundersen V. (2005). Major and trace elements in organically or conventionally produced milk. J. Dairy Res..

[36] Zhai X.W., Zhang Y.L., Qi Q., Bai Y., Chen X.L., Jin L.J., Ma X.G., Shu R.Z., Yang Z.J., Liu F.J. (2013). Effects of molybdenum on sperm quality and testis oxidative stress. Syst. Biol. Reprod. Med..

[37] Wang H.W., Zhou B.H., Zhang S., Guo H.W., Zhang J.L., Zhao J., Tian E.J. (2016). Reproductive toxicity in male mice after exposure to high molybdenum and low copper concentrations. Toxicol. Ind. Health.

[38] Trumbo P., Yates A.A., Schlicker S., Poos M. (2001). Dietary Reference Intakes: Vitamin A, Vitamin K, Arsenic, Boron, Chromium, Copper, Iodine, Iron, Manganese, Molybdenum, Nickel, Silicon, Vanadium, and Zinc. J. Acad. Nutr. Diet..

[39] Fieve R.R., Meltzer H.L. (1974). Proceedings: Rubidium salts--toxic effects in humans and clinical effects as an antidepressant drug. Psychopharmacol. Bull..

[40] Meltzer H.L., Fieve R.R., Essman W.B., Valzelli L. (1975). Rubidium in psychiatry and medicine: An overview. Current Developments in Psychopharmacology.

[41] Placidi G., Lenzi A., Lazzerini F., Dell’Osso L., Cassano G.B., Akiskal H.S. (1988). Exploration of the clinical profile of rubidium chloride in depression: A systematic open trial. J. Clin. Psychopharmacol..

[42] Brundusino A.O., Cairoli S. (1996). The pharmacological action of rubidium chloride in depression. Minerva Psichiatr..

[43] Chellan P., Sadler P.J. (2015). The elements of life and medicines. Philos. Trans. R. Soc. A.

[44] Osacky M., Geramian M., Ivey D.G., Liu Q., Etsell T.H. (2015). Influence of Nonswelling Clay Minerals (Illite, Kaolinite, and Chlorite) on Nonaqueous Solvent Extraction of Bitumen. Energy Fuels.

[45] Campbell L.M., Fisk A.T., Wang X., Köck G., Muir D.C.G. (2005). Evidence for biomagnification of rubidium in freshwater and marine food webs. Can. J. Fish. Aquat. Sci..

[46] National Research Council (1983). Selenium in Nutrition: Revised Edition. https://www.ncbi.nlm.nih.gov/books/NBK216727/pdf/Bookshelf_NBK216727.pdf.

[47] Gaucheron F., Park Y.W., Haenlein G.F.W. (2013). Milk minerals, trace elements, and macroelements. Milk and Dairy Products in Human Nutrition: Production, Composition and Health.

[48] Yanardag R., Orak H. (1999). Selenium content of milk and milk products of Turkey II. Biol. Trace Elem. Res..

[49] Agency for Toxic Substances and Disease Registry (2004). Toxicological Profile for Strontium. https://www.atsdr.cdc.gov/toxprofiles/tp159.pdf.

[50] Emsley J. (2011). An A–Z guide to the elements. Nature’s Building Blocks.

[51] Dudas M.J., Pawluk S. (1977). Heavy metals in cultivated soils and in cereal crops in Alberta. Can. J. Soil Sci..

[52] Tchounwou P.B., Yedjou C.G., Patlolla A.K., Sutton D.J. (2012). Heavy metals toxicity and the environment. Molecular, Clinical and Environmental Toxicology.

[53] Smedley P.L., Kinniburgh D.G. (2002). A review of the source, behaviour and distribution of arsenic in natural waters. Appl. Geochem..

[54] Lemay T.G. Arsenic Concentrations in Quaternary Drift and Quaternary-Tertiary Buried Channel Aquifers in the Athabasca Oil Sands (In Situ) Area, Alberta—EUB/AGS Geo-Note; Alberta Energy and Utilities Board: 2002; Volume 41. https://ags.aer.ca/publications/GEO_2002_04.html.

[55] Anderson R.A., Polansky M.M., Bryden N.A., Canary J.J. (1991). Supplemental-chromium effects on glucose, insulin, glucagon, and urinary chromium losses in subjects consuming controlled low-chromium diets. Am. J. Clin. Nutr..

